# A Modified Multiaxial Fatigue Model and Its Application for the Fatigue Life Prediction of Aircraft Hydraulic Pipes

**DOI:** 10.3390/ma17246154

**Published:** 2024-12-17

**Authors:** Yantian Wang, Yuanying Qiu, Jing Li, Jin Bai, Yan Wang

**Affiliations:** 1 School of Mechatronic Engineering, Xidian University, No. 2 South Taibai Road, Xi’an 710071, China; 2 Xi’an Aerospace Propulsion Test Technology Institute, Xi’an 710100, China

**Keywords:** multiaxial fatigue, aircraft hydraulic pipe, power spectral density, critical plane theory, fatigue life prediction

## Abstract

The fatigue failure of a structure may occur under a multiaxial vibration environment; it is necessary to establish a better multiaxial fatigue life prediction model to predict the fatigue life of the structure. This study proposes a new model (MWYT) by introducing the maximum absolute shear stress into the WYT model. The feasibility of the MWYT model is verified by using the multiaxial fatigue experimental data of 304 stainless steel, Q235B steel, 7075-T651 aluminum alloy and S355J0 steel. Further, finite element vibration simulations are performed on a typical parallel hydraulic pipe structure, and the vibration simulation results of the pipe structure are verified through the vibration experiment. Finally, the MWYT model is used to predict the fatigue lives of the pipe structure under random excitation and pulsation excitation, respectively, and the fatigue life of the pipe structure under the combined loading from random excitation and pulsation excitation is predicted based on Miner’s rule. By comparing with the design life of the aircraft, the predicted life of the pipe structure meets the service requirements for it.

## 1. Introduction

With the continuous developments of various industries, many metallic materials are widely used in automobiles, aircraft, and marine ships and other industries to manufacture various structural components [1,2,3]. While fatigue and reliability problems of structures have gradually been exposed under multiaxial loading [4,5,6,7,8], some structural components such as hydraulic pipeline systems, flaps and engine shafts are subjected to proportional and nonproportional multiaxial loadings, which may cause structural failure due to multiaxial fatigue [9,10,11]. According to the damage mechanism of multiaxial fatigue, the fatigue life of an structure is not only affected by the material properties and the magnitude of the external loading, but also by the loading path of the external loading and the intensity of the material’s response to different loading paths [12,13]. Considering the failure behaviors of materials under various loadings, multiaxial fatigue theoretical models have been developed [14,15,16,17,18].

After decades of research, some multiaxial fatigue life prediction models have been proposed by scholars in the field of fatigue. Several typical models are as follows. Smith et al. [19] proposed a multiaxial fatigue model, using the product of the maximum normal stress and normal strain amplitude on the plane, where the maximum amplitude of the normal strain is located to establish a multiaxial fatigue life model. Fatemi et al. [20] believed that the maximum shear strain controlled the crack initiation process, and the normal stress should be introduced in the damage parameters to consider the additional strengthening phenomenon of the nonproportional loading cycle. In addition, they proposed the use of the amplitude of the shear strain and the maximum normal stress on the critical plane as the fatigue damage parameters. Shang et al. [21] considered that the maximum amplitude of the shear strain and the range of the normal strain on the critical plane are important damage parameters, then combined the amplitude of the shear strain and the range of the normal strain into an equivalent parameter and proposed a fatigue model independent of loading paths. The models proposed in Refs. [19,20,21] did not simultaneously consider the influences of the shear and normal strains, shear and normal stresses on fatigue damage, all of which have poorer prediction effects on specimens under constant amplitude loading with non-zero mean normal and shear stresses [22,23]. In order to fully consider the influences of various variables on the critical plane for fatigue damage, Wang et al. [22] took the plane with the maximum amplitude of the shear strain as the critical plane, and then proposed a new multiaxial fatigue model (WYT). The amplitude of the shear strain, range of the normal strain, maximum shear and maximum normal stresses were introduced into the WYT model so that it could consider the influences of strains and stresses on fatigue damage. However, some studies have shown that both positive and negative mean shear stresses on the critical plane reduce the specimen life under constant amplitude loading with non-zero mean normal and shear stresses [23,24,25], which was not considered in the WYT model. If the WYT model is modified, its life prediction effect on the specimen may be further improved. Therefore, a modified multiaxial fatigue model is proposed based on the existing model in this study, and applied to the fatigue life prediction of the aircraft hydraulic pipe structure.

As an important part of the aircraft, hydraulic pipes are simultaneously subjected to multiaxial random excitation and pulsation excitation [9], which may easily cause multiaxial fatigues of pipe structures; thus, aircraft designers are particularly concerned about pipe structure problems. There are some multiaxial fatigue experimental data available in Refs. [24,26,27,28], which can be used to verify the life prediction effect of the proposed multiaxial fatigue model on the specimen. Furthermore, using the modified multiaxial fatigue model to predict the fatigue life of the hydraulic pipe structure is a significant engineering application.

## 2. Determination of the Critical Plane

Under multiaxial strain-controlled loading, a point strain state at the thin-walled tube is expressed by [29,30]:

(1) $$\varepsilon =\left(\begin{array}{ccc} \varepsilon_{x} & \frac{\gamma_{xy}}{2} & 0\\ \frac{\gamma_{yx}}{2} & -\upsilon_{eff}\varepsilon_{x} & 0\\ 0 & 0 & -\upsilon_{eff}\varepsilon_{x} \end{array}\right)$$

where $\varepsilon_{x}$ and $\gamma_{xy}$ are the normal and shear strains, respectively, and $\gamma_{xy}=\gamma_{yx}$; $\upsilon_{eff}$ is the effective Poisson’s ratio, and $\upsilon_{eff}=0.5-\frac{(0.5-\upsilon_{e})\Delta \sigma_{eq,a}}{E\Delta \varepsilon_{eq,a}}$ [29,30]. Among them, $\upsilon_{e}$, *E*, $\Delta \sigma_{eq,a}$ and $\Delta \varepsilon_{eq,a}$ are the elastic Poisson’s ratio, elastic modulus, equivalent stress amplitude, and equivalent strain amplitude, respectively.

Assuming that the axial and shear strains are sine waves, then

(2) $$\varepsilon_{x}(t)=\varepsilon_{x,a}\sin\omega t+\varepsilon_{x,m}$$

(3) $$\gamma_{xy}(t)=\gamma_{xy,a}\sin(\omega t-\varphi )+\gamma_{xy,m}$$

where φ is the phase angle between the axial and shear strains. $\varepsilon_{x,a}$ and $\gamma_{xy,a}$ are the amplitudes of the axial and shear strains, respectively. $\varepsilon_{x,m}$ and $\gamma_{xy,m}$ are the mean values of the axial and shear strains, respectively.

The amplitudes of the shear and normal strains on the plane with the maximum shear strain which makes an angle $\theta_{c}$ with the thin-walled tube axis can be expressed as [31]:

(4) $$\frac{\Delta \gamma_{\max}}{2}=\varepsilon_{x,a}{\left\{{\left[\lambda \cos(2\theta_{c})\cos\varphi -(1+\upsilon_{eff})\sin(2\theta_{c})\right]}^{2}+{\left[\lambda \cos(2\theta_{c})\sin\varphi \right]}^{2}\right\}}^{0.5}$$

(5) $$\frac{\Delta \varepsilon_{n}}{2}=\frac{\varepsilon_{x,a}}{2}{\left\{{\left[2(1+\upsilon_{eff})\cos^{2}\theta_{c}+\lambda \sin(2\theta_{c})\cos\varphi -2\upsilon_{eff}\right]}^{2}+{\left[\lambda \sin(2\theta_{c})\sin\varphi \right]}^{2}\right\}}^{0.5}$$

where

(6) $$\theta_{c}=\frac{1}{4}\tan^{-1}\left[\frac{2\lambda (1+\upsilon_{eff})\cos\varphi }{{\left(1+\upsilon_{eff}\right)}^{2}-\lambda^{2}}\right]$$

(7) $$\lambda =\frac{\gamma_{xy,a}}{\varepsilon_{x,a}}$$

The plane with the maximum amplitude of the shear strain is considered to be the critical plane when two or more planes have the same maximum amplitude of the shear strain, by taking the plane with the maximum range of the normal strain as the critical plane. Refs. [23,32,33] show the calculation process of different variables on each plane.

## 3. The Modified WYT Model and Its Application

### 3.1. A Modified Multiaxial Fatigue Model

In order to fully consider the influences of strains and stresses on the plane on fatigue damage, Wang et al. [22] took the plane with the maximum amplitude of the shear strain as the critical plane, and then proposed a multiaxial fatigue model (WYT). The normal strain range $\Delta \varepsilon_{n}$, maximum shear strain amplitude $\Delta \gamma_{\max}/2$, normal stress correction factor $\sigma_{n,\max}/\sigma_{f}^{\prime }$ and shear stress correction factor $\tau_{\max}/\tau_{f}^{\prime }$ are introduced into the WYT model to consider the influences of strains and stresses. The expression of the WYT model is as follows [22]:

(8) $$\frac{\Delta \gamma_{\max}}{2}\left(1+\frac{\tau_{\max}}{\tau_{f}^{\prime }}\right)+\Delta \varepsilon_{n}\left(1+\frac{\sigma_{n,\max}}{\sigma_{f}^{\prime }}\right)=f\left(N_{f}\right)$$

where $\tau_{\max}$ and $\sigma_{n,\max}$ are the maximum shear and maximum normal stresses on the critical plane, respectively; $N_{f}$ is the fatigue life; $\tau_{f}^{\prime }$ and $\sigma_{f}^{\prime }$ are the shear fatigue strength coefficient and fatigue strength coefficient, respectively.

Some studies have shown that whether the mean shear stress on the critical plane is positive or negative, it reduces the life of the specimen [23,24,25]. The WYT model did not consider this influence, so this study modifies the WYT model through changing the maximum stress $\tau_{\max}$ in the WYT model to the maximum absolute shear stress ${\left|\tau \right|}_{\max}$, i.e.,

(9) $$\frac{\Delta \gamma_{\max}}{2}\left(1+\frac{{\left|\tau \right|}_{\max}}{\tau_{f}^{\prime }}\right)+\Delta \varepsilon_{n}\left(1+\frac{\sigma_{n,\max}}{\sigma_{f}^{\prime }}\right)=f\left(N_{f}\right)$$

where ${\left|\tau \right|}_{\max}$ is the maximum absolute shear stress on the critical plane.

In this study, the modified model Equation (9) is called the MWYT model.

### 3.2. Experimental Data Verification

#### 3.2.1. Experimental Data Selection

**Figure 1:**
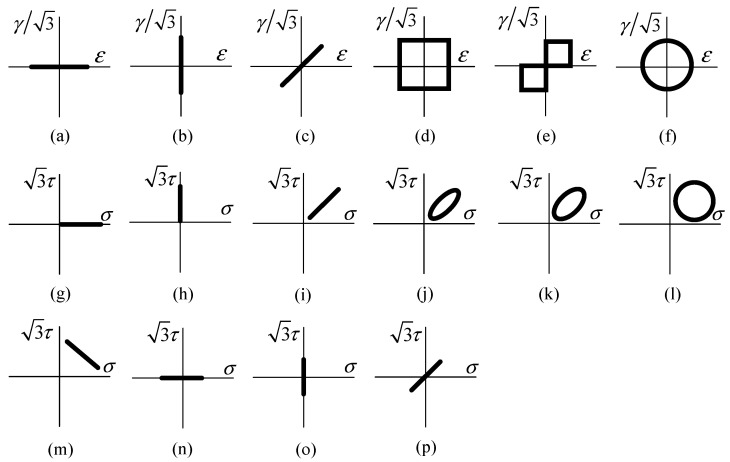
Loading paths. (**a**,**n**) are axial loading; (**b**,**o**) are torsional loading; (**c**,**p**) are proportional loading. For of them, φ = 0°; (**d**–**f**) are non-proportional loading, φ = 90° in paths (**d**,**f**), φ = 45° in path (**e**); (**g**) is axial loading with not-zero mean normal stress; (**h**) is torsional loading with non-zero mean shear stress; (**i**) is proportional loading with non-zero mean normal and shear stresses, φ = 0°; (**j**–**m**) are non-proportional loading with non-zero mean normal and shear stresses. φ is 30°, 45°, 90° and 180° respectively. Among them, (**a**–**f**) are strain controlled loading, (**g**–**p**) are stress controlled loading.

To verify the prediction effects of the MWYT model on different material specimens, this study selects multiaxial fatigue experimental data of four materials [24,26,27,28]. Meanwhile, the life prediction effects of the MWYT model on various materials are compared with those of the SDG and WYT models. Here, the SDG model means the multiaxial fatigue model proposed by Shang [21]. The multiaxial fatigue experimental data of 304 steel are taken from Ref. [26] and the loading paths are shown in Figure 1a–f; the multiaxial fatigue experimental data of Q235B steel are taken from Ref. [27] and the loading paths are shown in Figure 1a–c,f; the multiaxial fatigue experimental data of 7075-T651 aluminum alloy are taken from Ref. [24] and the loading paths are shown in Figure 1a,b,g–m; the multiaxial fatigue experimental data of S355J0 steel are taken from Ref. [28] and the loading paths are shown in Figure 1g–i,n–p. 

The detailed experimental processes of four materials were described, respectively, in Refs. [24,26,27,28]. The fatigue properties of these materials are shown in Table 1.

#### 3.2.2. Fatigue Life Prediction of Four Material Specimens

**Figure 2:**
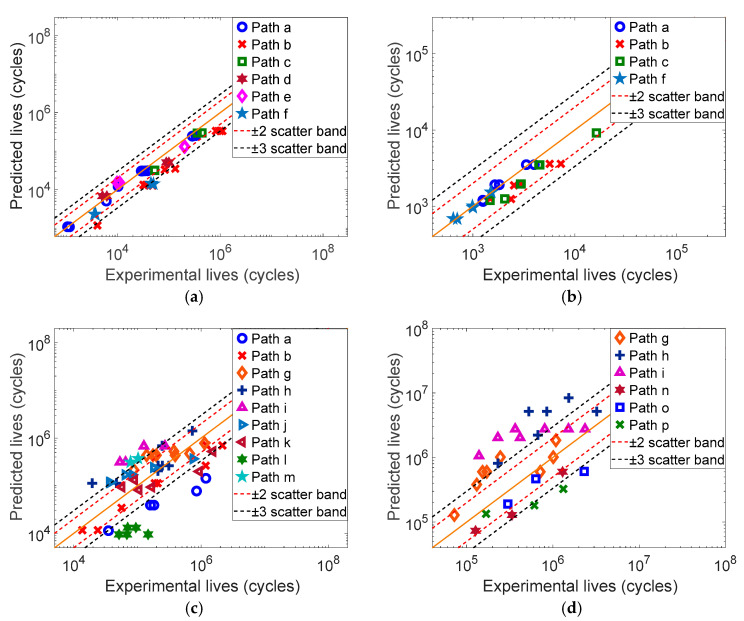
Predicted results of the SDG model for four material specimens. (**a**) 304, (**b**) Q235B, (**c**) 7075-T651, (**d**) S355J0.

**Figure 3:**
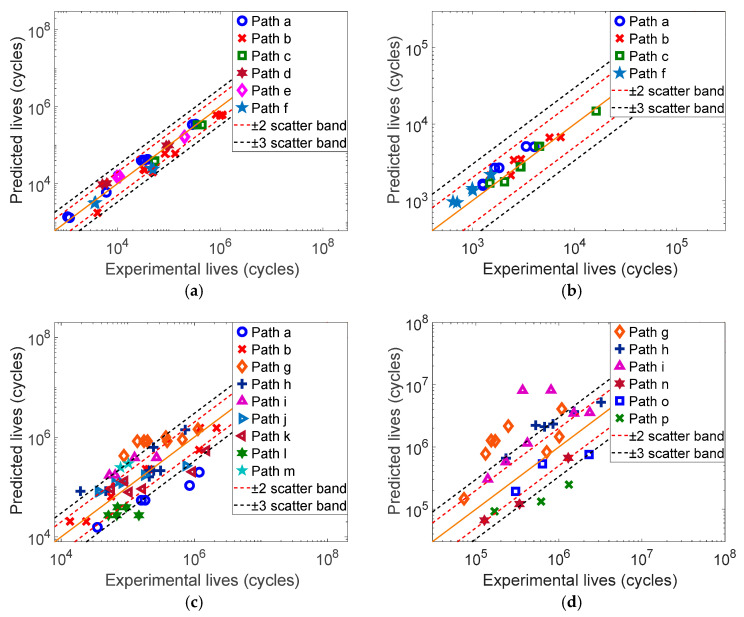
Predicted results of the WYT model for four material specimens. (**a**) 304, (**b**) Q235B, (**c**) 7075-T651, (**d**) S355J0.

**Figure 4:**
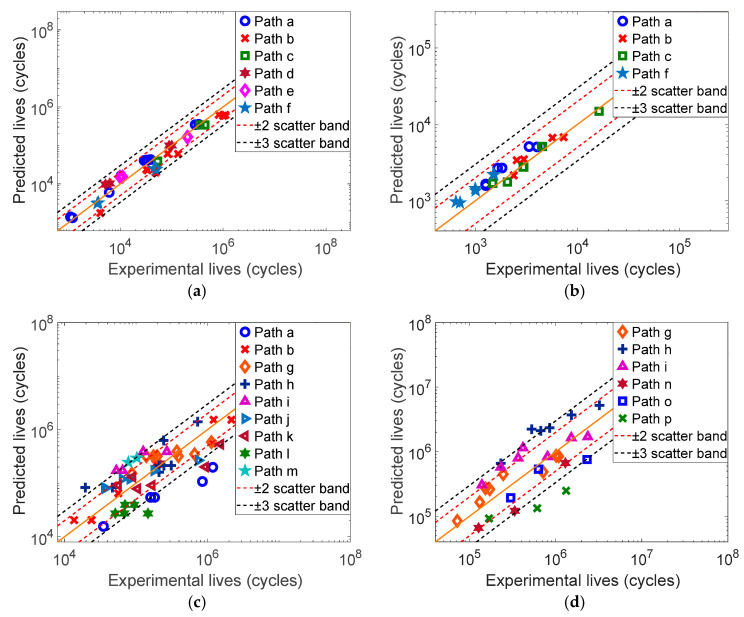
Predicted results of the MWYT model for four material specimens. (**a**) 304, (**b**) Q235B, (**c**) 7075-T651, (**d**) S355J0.

The mechanical parameters of each material are shown in Table 1. Based on the multiaxial fatigue experimental data in Refs. [24,26,27,28], the fatigue life of each material specimen under constant amplitude loading is predicted by using the SDG, WYT and MWYT models, respectively, and the prediction results are shown in Figure 2, Figure 3 and Figure 4.

According to Figure 2, we can see that the SDG model has better prediction effect on 304, with only a few data points outside the three times error band. Meanwhile, the prediction results of the SDG model for Q235 are all within the three times error band, and are mainly concentrated within the two times error band. However, the SDG model has poorer prediction effects on 7075-T651 and S355J0, with nearly half of the data points outside the three times error band.

Based on Figure 3, the prediction results of the WYT model for 304 are all within the three times error band, and the prediction results for Q235B are all within the two times error band. The WYT model also has better prediction effect on 7075-T651, and most of the prediction results are within the three times error band. However, the WYT model has poorer prediction effect on S355J0, and some data points are outside the three times error band.

From Figure 4, the prediction results of the MWYT model for 304 are all within the three times error band, and the prediction results for Q235B are all within the two times error band. The MWYT model also has better prediction effects on 7075-T651 and S355J0, and most of the prediction results of the two materials are within the three times error band.

This study counts the distributions of each model’s prediction results for four materials in the error bands, as shown in Table 2. From Table 2, the prediction effects of the MWYT model for four materials are better than those of the SDG and WYT models.

For 304 and Q235B, the mean shear and mean normal stresses on all loading paths are zero, and the lives of the specimens are not influenced by mean stresses. Therefore, the WYT and MWYT models have the same effect on the life prediction of 304 and Q235B. Meanwhile, the SDG model also has better prediction effects on the two materials.

Since the mean stresses of the 7075-T651 and S355J0 specimens under constant amplitude loading are not zero, the lives of the specimens are influenced by mean stresses. Furthermore, the maximum shear stress $\tau_{\max}$ and the maximum absolute shear stress ${\left|\tau \right|}_{\max}$ on the critical plane are sometimes not equal, so the prediction effects of the WYT and the MWYT models for the 7075-T651 and S355J0 specimens are different. Studies have shown that whether the mean shear stress on the critical plane is positive or negative, it reduces the life of the specimen [23,24,25]. The MWYT model considers this influencing factor, so its prediction effects on 7075-T651 and S355J0 are better than those of the WYT models. The SDG model only considers the influences of the normal and shear strains on the critical plane on fatigue damage, but does not consider the influences of the mean normal and shear stresses, and it has poorer prediction effects on 7075-T651 and S355J0.

Based on the above analysis, the order of the effects of the three models on fatigue life prediction for various materials is as follows: MWYT, WYT and SDG.

## 4. Fatigue Life Prediction of the Parallel Aircraft Hydraulic Pipe Structure Under Combined Loading

As an important part of the aircraft, hydraulic pipes are often subjected to random excitation from the engine and pulsation excitation from the hydraulic oil, both of which cause pipe vibration. The vibration fatigue problems of pipes have always been the focus of aircraft engineers. Therefore, predicting the fatigue life of the aircraft hydraulic pipe structure has certain engineering significance. In this section, the MWYT model is used to predict the fatigue life of a typical parallel hydraulic pipe structure under combined loading.

**Figure 5:**
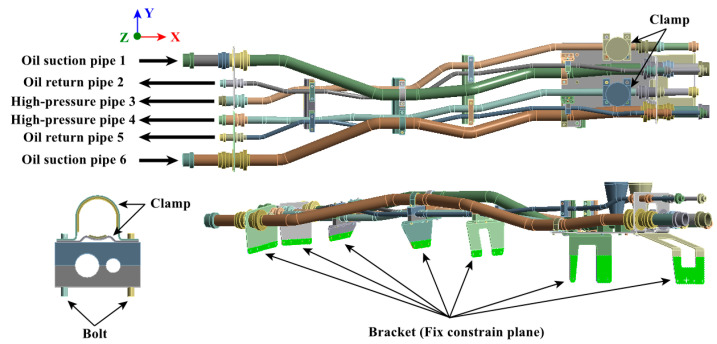
Hydraulic pipe structure.

**Figure 6:**
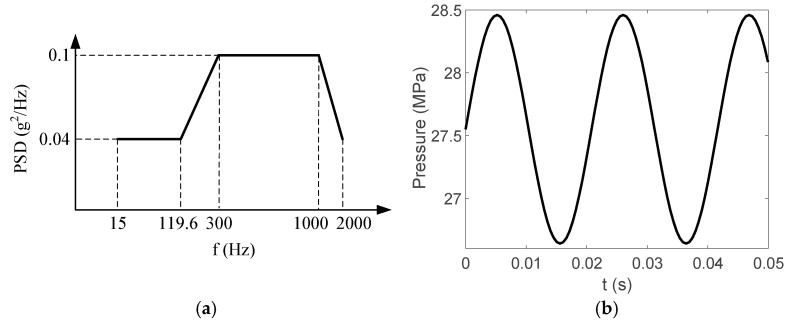
Pipe structure excitations. (**a**) Random excitation acceleration PSD. (**b**) Hydraulic oil pulsation function curve.

The structure of the hydraulic pipe structure is shown in Figure 5. The density of the hydraulic oil is 850 kg/m^3^, and the material mechanical parameters of each pipe component are shown in Table 3. The pipe structure is simultaneously subjected to random excitation from the engine and pulsation excitation from the hydraulic oil. The power spectrum density (PSD) of the acceleration is shown in Figure 6a, and the pulsation function of the hydraulic oil is shown in Figure 6b.

### 4.1. Simulation of the Pipe Structure Under Random Excitation and Pulsation Excitation

#### 4.1.1. Random Vibration Simulation of the Pipe Structure

**Figure 7:**
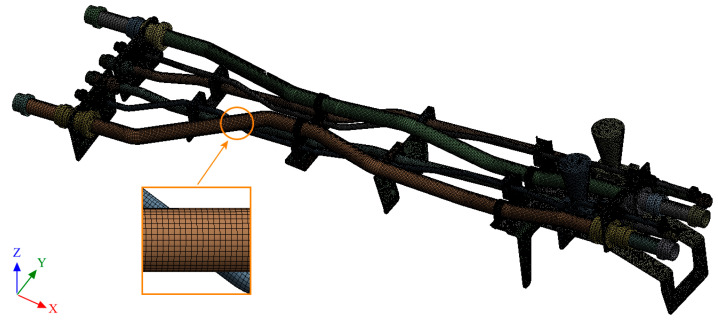
Finite element model of the pipe structure.

**Figure 8:**
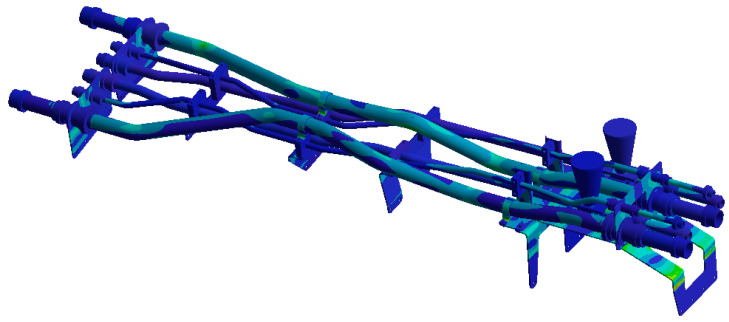
Stress cloud diagram under random excitation.

The finite element model is established on the pipe structure shown in Figure 7. The random acceleration excitation with its acceleration PSD shown in Figure 6a is applied to the pipe structure in the Z direction. The stress cloud diagram obtained from the simulation is shown in Figure 8, and the maximum stress value and stress cloud diagram of each of the same material components are extracted as shown in Table 4 (the maximum stress points are numbered A1-A6). According to Table 4, the maximum 3*σ* stress of each component does not exceed the yield strength of the material, and which means that no strength failure will occur.

#### 4.1.2. Pulsation Excitation Simulation of the Pipe Structure

**Figure 9:**
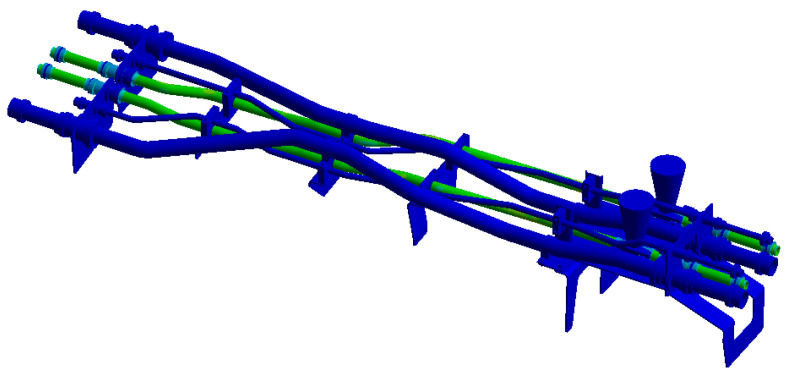
Stress cloud diagram under pulsation excitation.

Since the oil in the return pipe or suction pipe is quasi-static, and the oil pressure is less than 2 MPa, the fluid–structure coupling is ignored. The pulsation excitation function with a mean pressure of 28 MPa is set for the oil in the two high-pressure pipes, as shown in Figure 6b. The simulated cloud diagram is shown in Figure 9, and the extracted stress cloud diagram and maximum stress of the same material component are shown in Table 5 (the maximum stress points are numbered B1-B6).

According to Table 5, the maximum stress of each component does not exceed the yield strength of the material, which does not cause the strength failure of the structure. The stresses of the two high-pressure pipes are obviously higher than those of other components, which are also consistent with the actual stress conditions of the pipe structure.

### 4.2. Experimental Comparison Validation

#### 4.2.1. Random Excitation Experiment

(1) Experiment description

**Figure 10:**
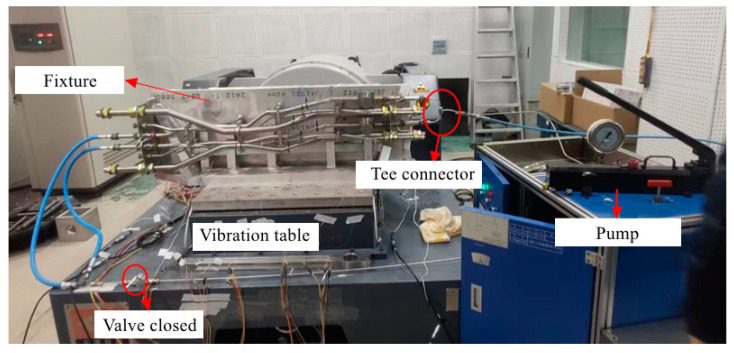
Random excitation experiment.

**Figure 11:**
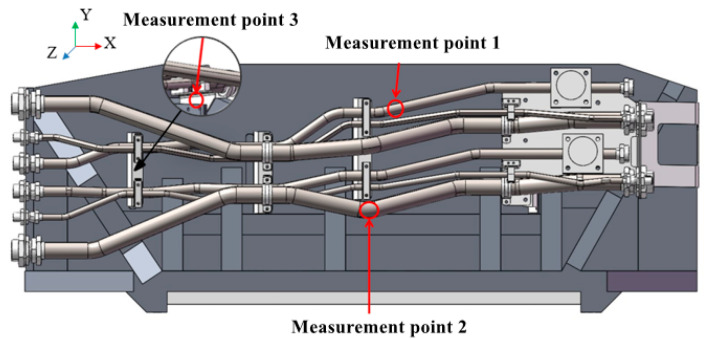
Location of the measurement points for random excitation experiment.

The physical connection of the random excitation experiment is shown in Figure 10. The pipes are fixed on the shaking table through the fixture, and the high-pressure pipe is connected with the hydraulic pump. The acceleration PSD applied during the experiment is shown in Figure 6a. The strain gauges are set at the three measurement points shown in Figure 11.

(2) Comparison of experiment and simulation

**Figure 12:**
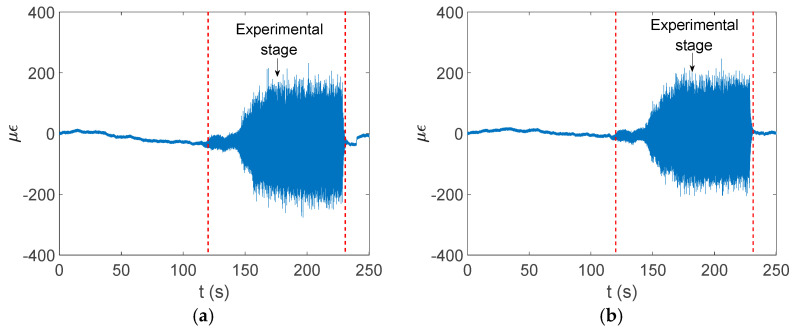
Strain signal acquisition at pipe measurement point 2. (**a**) Axial strain signal. (**b**) Circumferential strain signal.

**Figure 13:**
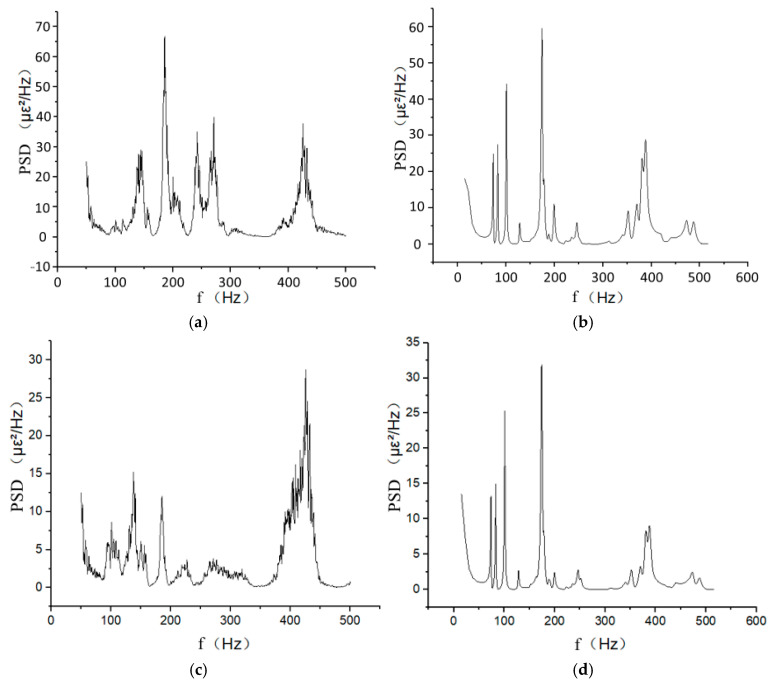
Comparison of strain PSD for experiment and simulation at measurement point 2. (**a**) FFT signal of experimental axial strain. (**b**) Simulated axial strain PSD. (**c**) FFT signal of experimental circumferential strain. (**d**) Simulated circumferential strain PSD.

The strain response signal of measurement point 2 is obtained as shown in Figure 12. Since the experimental random strain signal is shown in the time domain, it is not conducive to compare with the frequency domain simulation results. Strain signals in the experimental stage are converted into the frequency domain by fast Fourier transform (FFT). Strain PSD at the same location is extracted by simulation and compared with it, as shown in Figure 13.

Based on Figure 13, the experimental results from measurement point 2 show that the pipe structure has vibration peaks near four frequency points: 140 Hz, 184 Hz, 240 Hz to 270 Hz and 425 Hz. The simulation results show vibration peaks near four frequency points: 125 Hz, 178 Hz, 246 Hz and 378 Hz. The relative error between each pair of frequency points is within 20%.

The strain PSD root mean square (RMS) value of measurement point 2 is compared with the simulation results as shown in Table 6; it can be seen that the simulation results are close to the experimental results. Among them, the maximum error is 20.8%, within the engineering allowable range.

The data analysis processes of measurement points 1 and 3 are similar to that of measurement point 2. Due to the limited length, they are not described in detail here.

#### 4.2.2. Pulsation Excitation Experiment

(1) Experiment description

**Figure 14:**
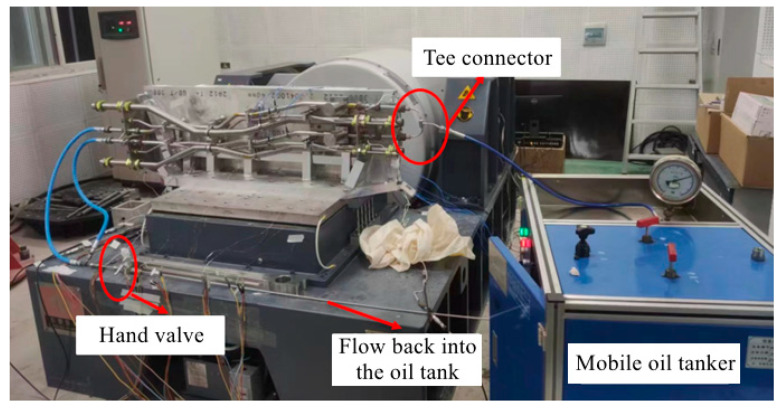
Pulsation experiment.

During the experiment, a mobile oil truck is used to input the pulsating pressure oil into the high-pressure pipe. Passing through the high-pressure pipe, the oil flows through the tee connector, and then returns to the oil tank through the switch acting as the load, as shown in Figure 14. The return pipe and suction pipe are filled with static oil. The oil pulsation excitation function of the experimental plunger pump is shown in Figure 6b.

**Figure 15:**
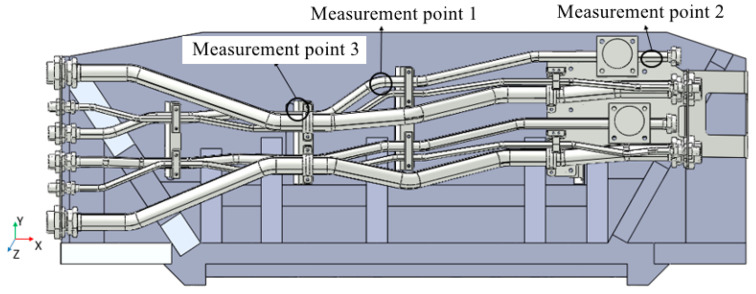
Location of measurement points for pulsation excitation experiment.

Axial and circumferential strain gauges are installed at the three locations in the pipe system model shown in Figure 15.

(2) Comparison of the experiment and the simulation

**Figure 16:**
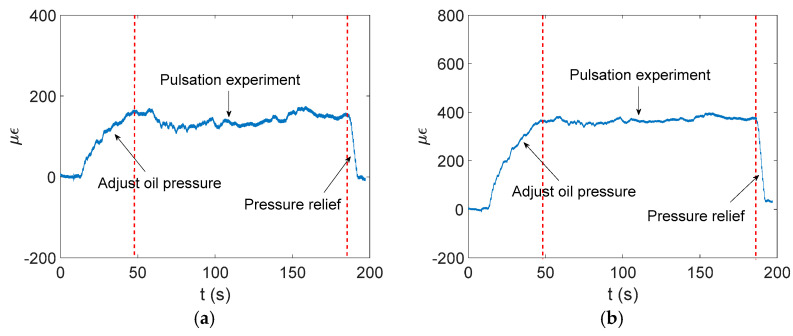
Strain signal acquisition at pipe measurement point 2. (**a**) Axial strain signal. (**b**) Circumferential strain signal.

**Figure 17:**
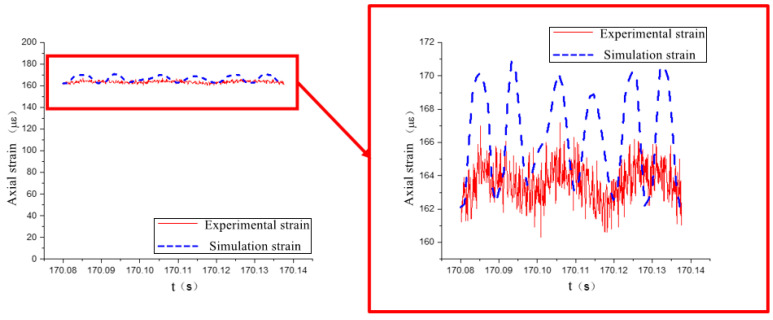
Comparison of axial strain data at measurement point 2.

**Figure 18:**
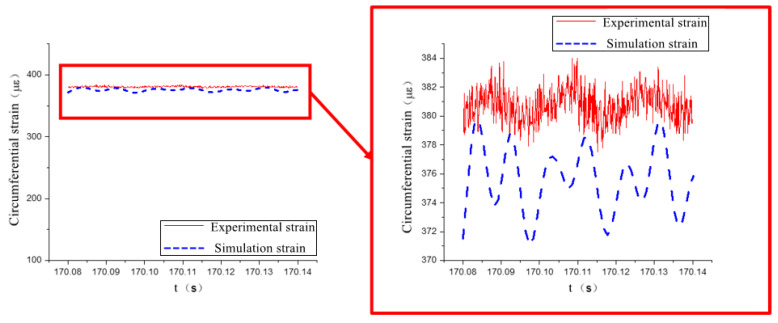
Comparison of circumferential strain data at measurement point 2.

The experimental data show that the circumferential strain is more obvious than the axial strain. The time domain signal collected by the strain gauge at measurement point 2 is shown in Figure 16, and the finite element simulation calculation results at measurement point 2 are extracted and compared with the experiment, as shown in Figure 17 and Figure 18.

As shown in Figure 17 and Figure 18, the simulation results of measurement point 2 are in good agreement with the experimental data. In the axial direction, the mean value of the experimental strain data is 163.3 με, and the mean value of the simulation strain data is 166.1 με, and the relative error between the two is within 2%. In the circumferential direction, the mean value of the experimental strain data is 380.8 με, and the mean value of the simulation strain data is 375.8 με, and the relative error between the two is within 1.5%.

The data analysis processes of measurement points 1 and 3 are similar to that of measurement point 2. Due to the limited length, they are not described in detail here.

There are some reasons for the errors between the simulation results and experimental results, such as the fact that the directions of the strain gauges may not be completely axial and circumferential, while the simulation extraction results are exactly along the axial and circumferential directions of the pipe. There are frictions among the contacted components in the vibration experiment, while in the simulation, the contacts among the components are all set as binding, and the binding contact assumed by the simulation makes the constraints between the contact surfaces tighter than in reality. There are also dimensional errors between the experimental model and the simulation model. The errors of each measurement point in the vibration experiment are basically within 20%, and the effectiveness of the simulation method is verified.

### 4.3. Fatigue Life Prediction of the Pipe Structure

By comparing the experimental results with the simulation results, it is proved that the simulation of the hydraulic pipe structure is feasible. According to Table 4 and Table 5, the fatigue failure of the pipe structure does not occur whether under random excitation or pulsation excitation. In this section, the fatigue life of the pipe structure is predicted by the MWYT model. The fatigue parameters of each material are shown in Table 7.

**Figure 19:**
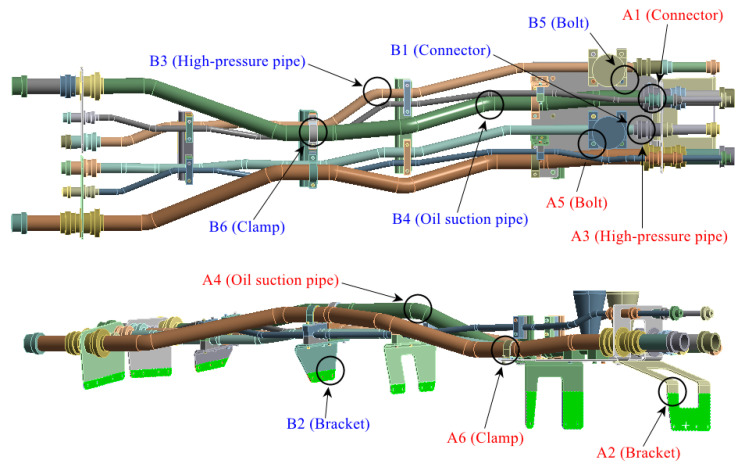
Locations of critical points.

To visually indicate the position of each critical point (maximum stress point), the critical point positions in Table 4 and Table 5 are marked in the pipe model as shown in Figure 19. It is known that each critical point does not coincide, the life of each critical point is predicted for safety reasons, and the minimum life is considered as the final life of the pipe structure.

#### 4.3.1. Fatigue Life Prediction of the Pipe Structure Under Random Excitation

Under variable amplitude loading, the fatigue damage is shown as follows [35]:

(10) $$D_{r}=\sum_{i=1}^{n}\frac{1}{N_{fi}}$$

where *n* and $N_{fi}$ are the number of cycles and the fatigue life corresponding to the *i*-th cycle, respectively.

Fatigue life is

(11) $$T_{r}=\frac{\Delta t_{r}}{D_{r}}$$

where $\Delta t_{r}$ is the duration of the time domain response spectral of the stress or strain.

When predicting the life of the specimen under random vibration loading in the frequency domain, if the stress and strain time histories of the critical point are unknown, first the stress and strain PSDs at the critical point are extracted, and then the stress–strain time history is obtained by IFFT. Second, the stress–strain time histories on different planes are obtained by coordinate transformation, and then the shear strain is counted by the cycle counting method, considering the plane with the maximum amplitude of the shear strain as the critical plane. Finally, the four-channel multiaxial cycle counting method [36] is used to count the variables on the critical plane and predict the fatigue life of the specimen using the MWYT model. The calculation process can also be found in Ref. [36].

**Figure 20:**
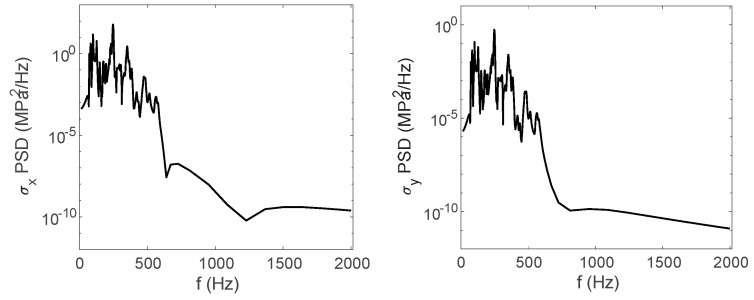
Stress PSDs in X and Y directions at point A1.

**Figure 21:**
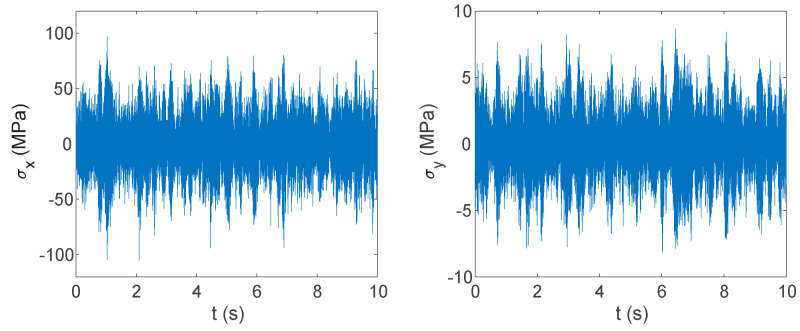
Stress time histories in X and Y directions at point A1.

The stress cloud diagrams of each component in Figure 19 have marked the locations of the maximum stress points and numbered the critical points. This section uses the MWYT model to predict the fatigue life of each component. Due to the limited length, only the stress PSDs in the X and Y directions at point A1 of the connector (seeing Table 4) are drawn in Figure 20. The sampling frequency is set to six times the highest excitation frequency (2000 Hz), and the sampling points total 120,000. By using the inverse fast Fourier transform (IFFT), the response time histories over 10 s are obtained in Figure 21.

The life prediction methods of the other component critical points are the same as that of point A1, so they are not described in detail here. The fatigue life of each component critical point is shown in Table 8. Among them, the minimum life of the critical point is 2.16 × 10^4^ h. It has been pointed out that the design service life of a military fighter aircraft is about 5000 h [37], so the predicted lives of all components can meet the requirements under random excitation.

#### 4.3.2. Fatigue Life Prediction of the Pipe Structure Under Pulsation Excitation

Under constant amplitude loading, the fatigue damage is shown as follows [35]:

(12) $$D_{p}=\frac{m}{N_{f}}$$

where $N_{f}$ and *m* are the fatigue life and the number of cycles of the constant amplitude signal, respectively.

Fatigue life is

(13) $$T_{p}=\frac{\Delta t_{p}}{D_{p}}$$

where $\Delta t_{p}$ is the duration of the time domain response spectral of the stress or strain.

For predicting the life of the specimen under constant amplitude loading in the time domain, the stress–strain time histories on different planes are obtained by coordinate transformation, and then the shear strain is counted by the cycle counting method, considering the plane with the maximum amplitude of the shear strain as the critical plane. Finally, the four-channel multiaxial cycle counting method [36] is used to count the variables on the critical plane, and the fatigue life of the specimen is predicted by using the MWYT model.

**Figure 22:**
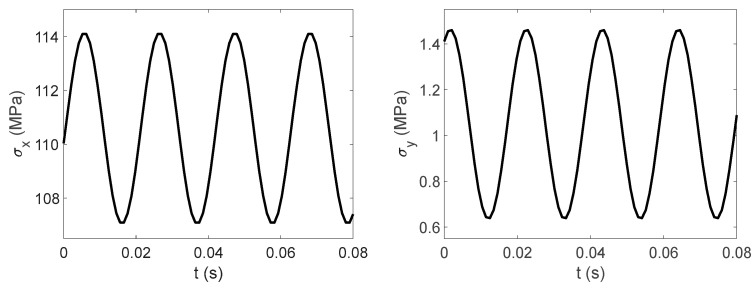
Stress time histories in X and Y directions at point B1.

This section uses the MWYT model to predict the fatigue life of each component in Figure 19. Due to the limited length, only the stable stress responses in the X and Y directions at point B1 are drawn in Figure 22.

This study takes the 10 s response time history of each variable, and uses the MWYT model to predict the fatigue life of each component. The fatigue life of each component critical point is shown in Table 9. Among them, the minimum life of the critical point is 6.19 × 10^12^ h. It has been pointed out that the design service life of military fighter aircraft is about 5000 h [35], so the predicted lives of all components can meet the requirements under pulsation excitation.

#### 4.3.3. Fatigue Life Prediction of the Pipe Structure Under Combined Excitation

In Section 4.3.1 and Section 4.3.2, the fatigue lives of the hydraulic piping structure under random excitation and pulsation excitation are calculated, respectively. This section predicts the fatigue life of the hydraulic piping structure under combined excitation of random and pulsation. According to the linear damage accumulation rule [35], the fatigue life under combined excitation is

(14) $$T_{total}=\frac{\Delta t}{D_{r}+D_{p}}$$

where Δt is the stress response time length, Δt=10 s.

The fatigue life of the pipe structure under combined excitation is calculated according to the data in Table 8 and Table 9, as shown in Table 10. Among them, the minimum life of the critical point is 2.15 × 10^4^ h. It has been pointed out that the design service life of military fighter aircraft is about 5000 h [35], so the predicted lives of all components can meet the requirements under combined excitation.

## 5. Conclusions

(1) The MWYT model is proposed by modifying the WYT model by changing the maximum shear stress $\tau_{\max}$ to the maximum absolute shear stress ${\left|\tau \right|}_{\max}$. The life prediction results on four material specimens show that the MWYT model has better effects than the SDG and WYT models. Among them, the prediction results of the MWYT for 304 are all within the three times error band, and the prediction results for Q235B are all within the two times error band, and most of the prediction results for 7075-T651 and S355J0 are within the three times error band.
(2) The dynamic simulation of a typical parallel hydraulic pipe structure is performed and compared with the experiment. By comparing the simulation strain results with the measured strain data, the finite element simulation is proved. Meanwhile, the simulation results show that the pipe structure does not undergo strength failure under either random excitation or pulsation excitation.
(3) The fatigue lives of the pipe structure under random excitation and pulsation excitation are predicted, respectively, and further, the fatigue life of the pipe structure under combined loading of random and pulsation excitations is predicted based on Miner’s rule. By comparing with the design life of the aircraft, the predicted life of the hydraulic pipe structure meets the requirement.

## Figures and Tables

**Table 1:** Material parameters.

Properties	304 [26]	Q235B [27]	7075-T651 [24]	S355J0 [28]
E (GPa)	183	204	71.7	213
G (GPa)	82.8	81.4	27.5	81.3
υ	0.117	0.3	0.306	0.31
$\sigma_{y}$ (MPa)	325	269	501	355
$\sigma_{f}^{\prime }$ (MPa)	1000	407.59	1235	880
$\varepsilon_{f}^{\prime }$	0.171	0.8091	0.243	0.126
*b*	−0.114	−0.0424	−0.138	−0.095
*c*	−0.402	−0.5827	−0.71	−0.448
$\tau_{f}^{\prime }$ (MPa)	709	398.13	797	508

**Table 2:** Distribution of the prediction results within the error bands for each material by using the SDG, WYT and MWYT models.

Scatter Band	Models	Materials			
		304	Q235B	7075-T651	S355J0
±3	SDG	82.86%	100%	57.69%	46.67%
	WYT	100%	100%	75%	60%
	MWYT	100%	100%	82.69%	83.33%
±2	SDG	68.57%	96%	36.54%	36.67%
	WYT	88.57%	100%	48.08%	30%
	MWYT	88.57%	100%	57.69%	56.67%

**Table 3:** Material properties of the pipe components.

Name	Material	ρ (kg/cm^3^)	E (GPa)	υ	$\sigma_{y}$ (MPa)	$\sigma_{u}$ (MPa)
Connector	0Cr15Ni5Cu4Nb	7800	190	0.24	200	≥930
Bracket	0Cr18Ni9	7850	199	0.3	205	520
High-pressure pipe	0Cr21Ni6Mn9N	7810	190	0.27	365	735
Oil return pipe						
Oil suction pipe	1Cr18Ni10Ti	7900	204	0.3	235	632
Buffer bottle	0Cr15Ni5Cu4Nb	7800	190	0.24	200	≥930
Bolt	30CrMnSiA	7750	196	0.3	835	1080
Clamp	1Cr18Ni9Ti	8030	206	0.3	200	550

**Table 4:** Maximum 3σ stress and its location of each component under random excitation.

Name	Maximum 3σ Stress (MPa)	Stress Cloud Diagram
Connector	46	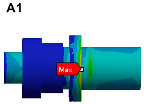
Bracket	103	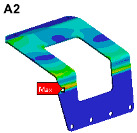
High-pressure pipeOil return pipe	83	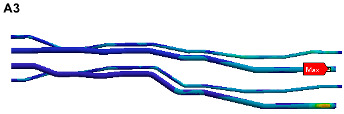
Oil suction pipe	107	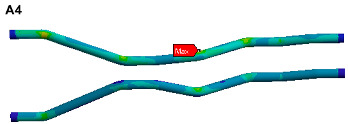
Bolt	221	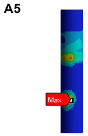
Clamp	171	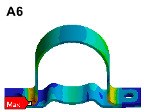

**Table 5:** Maximum stress value and location of each component under pulsation excitation.

Name	Maximum Stress (MPa)	Stress Cloud Diagram
Connector	116	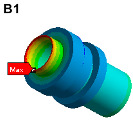
Bracket	126	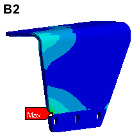
High-pressure pipe Oil return pipe	166	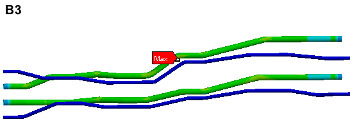
Oil suction pipe	6.9	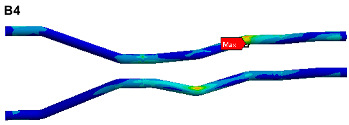
Bolt	70	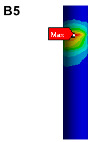
Clamp	20	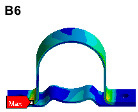

**Table 6:** Comparison of simulation strain data and experimental data at measurement point 2.

Directions	Experimental RMS Values (με)	Simulation RMS Values (με)	Relative Errors
Axial	57.37	45.42	20.8%
Circumferential	39.51	32.23	18.43%

**Table 7:** Material fatigue parameters of each component.

Name	Material	$\sigma_{f}^{\prime }$ (MPa)	$\varepsilon_{f}^{\prime }$	b	c	$\tau_{f}^{\prime }$ (MPa)
Connector	0Cr15Ni5Cu4Nb	1416	0.27	−0.09	−0.56	818
Bracket	0Cr18Ni9	880	0.4	−0.09	−0.56	508
High-pressure pipe Oil return pipe	0Cr21Ni6Mn9N	1164	0.3	−0.09	−0.56	672
Oil suction pipe	1Cr18Ni10Ti	1039	0.35	−0.09	−0.56	600
Bolt	30CrMnSiA	1612	0.25	−0.09	−0.56	931
Clamp	1Cr18Ni9Ti	927	0.38	−0.09	−0.56	535

Based on the Muralidharan–Manson method in Ref. [34], the fatigue parameters of each material are estimated.

**Table 8:** Fatigue lives of critical points under random excitation.

Critical Points	Damages	Lives (h)
A1	1.5 × 10^−13^	1.86 × 10^10^
A2	6.67 × 10^−9^	4.17 × 10^5^
A3	1.78 × 10^−16^	1.56 × 10^13^
A4	1.38 × 10^−10^	2.02 × 10^7^
A5	1.12 × 10^−22^	2.48 × 10^19^
A6	1.29 × 10^−7^	2.16 × 10^4^
B1	2.84 × 10^−16^	9.79 × 10^12^
B2	7.91 × 10^−11^	3.51 × 10^7^
B3	1.39 × 10^−23^	2.00 × 10^20^
B4	1.64 × 10^−13^	1.70 × 10^10^
B5	5.81 × 10^−24^	4.79 × 10^20^
B6	6.30 × 10^−8^	4.41 × 10^4^

**Table 9:** Fatigue lives of critical points under pulsation excitation.

Critical Points	Damages	Lives (h)
A1	4.85 × 10^−33^	5.73 × 10^29^
A2	2.77 × 10^−24^	1.00 × 10^21^
A3	1.88 × 10^−23^	1.47 × 10^20^
A4	1.02 × 10^−29^	2.73 × 10^26^
A5	7.67 × 10^−31^	3.63 × 10^27^
A6	2.18 × 10^−23^	1.27 × 10^20^
B1	1.58 × 10^−26^	1.76 × 10^23^
B2	4.49 × 10^−16^	6.19 × 10^12^
B3	6.04 × 10^−22^	4.60 × 10^18^
B4	9.69 × 10^−29^	2.87 × 10^25^
B5	5.15 × 10^−26^	5.39 × 10^22^
B6	5.18 × 10^−23^	5.37 × 10^19^

**Table 10:** Fatigue lives of critical points under combined excitation.

Critical Points	Damages	Lives (h)
A1	1.50 × 10^−13^	1.85 × 10^10^
A2	6.67 × 10^−9^	4.16 × 10^5^
A3	1.78 × 10^−16^	1.56 × 10^13^
A4	1.38 × 10^−10^	2.01 × 10^7^
A5	1.12 × 10^−22^	2.48 × 10^19^
A6	1.29 × 10^−7^	2.15 × 10^4^
B1	2.84 × 10^−16^	9.78 × 10^12^
B2	7.91 × 10^−11^	3.51 × 10^7^
B3	6.18 × 10^−22^	4.50 × 10^18^
B4	1.64 × 10^−13^	1.69 × 10^10^
B5	5.86 × 10^−24^	4.74 × 10^20^
B6	6.30 × 10^−8^	4.41 × 10^4^

## Data Availability

The raw data supporting the conclusions of this article will be made available by the authors on request. As the hydraulic pipe in the manuscript belongs to a certain type of military aircraft, more detailed information about the pipe cannot be provided for confidentiality reasons. Therefore, the data provided is limited.
